# Diverse Profiles of AI-1 Type Quorum Sensing Molecules in Cultivable Bacteria from the Mangrove (*Kandelia obovata*) Rhizosphere Environment

**DOI:** 10.3389/fmicb.2016.01957

**Published:** 2016-12-05

**Authors:** Zhi P. Ma, Yong M. Lao, Hui Jin, Guang H. Lin, Zhong H. Cai, Jin Zhou

**Affiliations:** ^1^The Division of Ocean Science and Technology, Graduate School at Shenzhen, Tsinghua UniversityShenzhen, China; ^2^Shenzhen Public Platform for Screening and Application of Marine Microbial ResourcesShenzhen, China

**Keywords:** quorum sensing, acyl homoserine lactone, diverse profiles, rhizobacteria, mangrove plant, plant-microbes interactions

## Abstract

Mangrove rhizosphere environment harbors diverse populations of microbes, and some evidence showed that rhizobacteria behavior was regulated by quorum sensing (QS). Investigating the diverse profiles of QS molecules in mangrove ecosystems may shed light on the bacterial roles and lead to a better understanding of the symbiotic interactions between plants and microbes. The aims of the current study focus on identifying AI-1 type QS signals, i.e., acyl homoserine lactones (AHLs), in *Kandelia obovata* rhizosphere environment. Approximately 1200 rhizobacteria were screened and 184 strains (15.3%) tested were positive. Subsequent 16s rRNA gene sequencing and dereplication analyses identified 24 species from the positive isolates, which were affiliated to three different phyla, including Proteobacteria, Firmicutes, and Actinobacteria. Thin-layer chromatography separation of extracts revealed diverse AHL profiles and detected at least one active compound in the supernatant of these 24 cultivable AHL-producers. The active extracts from these bacterial isolates were further evaluated by ultra performance liquid chromatography-mass spectrometry, and the carbon side chain length ranged from C4 to C14. This is the first report on the diversity of AI-1 type auto-inducers in the mangrove plant *K. obovata*, and it is imperative to expand our knowledge of plant-bacteria interactions with respect to the maintenance of wetland ecosystem health.

## Introduction

Wetland plants contain dense and abundant microbial communities within the thin-layer of root-adherent soil known as the rhizosphere environment (Bulgarelli et al., 2013). Rhizospheric bacteria are living in the direct vicinity of the roots, and root-associated bacteria play important roles in mangrove ecosystem involving nitrogen fixation (Alfaro-Espinoza and Ullrich, 2015), nutrient acquisition (Holguin et al., 2001; Berendsen et al., 2012; Prakash et al., 2015), abiotic stress tolerance (Rout and Southworth, 2013), as well as production of regulators of plant growth and development such as auxins, cytokinins, and gibberellins (Kaai et al., 2008). Additionally, the microbial communities associated with root also play an essential role in the matter cycle (for instance phosphorus, organic acids, and siderophores) and maintenance of the health of wetland ecosystem (Gomes et al., 2011; Zeng et al., 2014).

It is noteworthy that the structure and function of mangrove bacterial community determined by the interactions not only between bacterial groups but also between plants and bacteria (Berendsen et al., 2012). Plant-bacteria symbiosis is dependent on molecular and biochemical sensors to be able to dynamically perceive fluxes in their internal and external environments and coordinate the appropriate response at the whole ecosystem level (Scharf et al., 2016). Nevertheless, based on the extremely complex symbiotic relationship between plants and microbes, more work is still needed in order to better understand the important interactions between the inter- or intra-players of ecosystem function, plants and their associated microorganisms. Recent evidence showed that chemical ecology is a robust and efficient methodology to uncover the behavior and ecological functions of microbes (Schwartz et al., 2016).

Quorum sensing (QS) is a common form of chemical signaling receiving increasing attention from marine ecologists recently. QS is the coordinated regulation of gene expression in individual bacterial cells mediated by the population density-dependent release of extracellular signals (autoinducers, AI) to affect many diverse behaviors in a wide range of bacterial species (Banerjee and Ray, 2016). Microbial communities use a variety of molecules, including *N*-acyl-homoserine lactones (AHLs, AI-1), furanosyl borate (AI-2), polypeptides, and diketopiperazines, as autoinducing signals (Boyen et al., 2009; Rajput et al., 2016). Among them, AHLs are arguably the most common and important signaling molecules and regulate various biological functions including biofilm formation, genetic competence, symbiosis, motility, and the production of virulence factors in bacteria (especially in Gram-negative group; Atkinson and Williams, 2009; Hense and Schuster, 2015).

Based on the ecological importance of AHL signal, AHL production and distribution in various niches such as sediment, seaweed, coral, and marine snow have been extensively investigated in the field of marine microbiology (D’Angelo-Picard et al., 2005; Scott et al., 2006; DeAngelis et al., 2008; Ransome et al., 2014; Singh and Reddy, 2014; Certner and Vollmer, 2015; Jatt et al., 2015). At the same time, the ecological distribution and potential functions of AHL-producing microbes in plant field have also garnered much attention. AHL signaling has been shown to play a role in the manifestation of the plant-associated phenotypes of numerous autotrophic, pathogenic, and symbiotic bacterial strains (Chong et al., 2012; van Dam and Bouwmeester, 2016; Zhou et al., 2016). A recent study demonstrated that AHL-producing Alpha-proteobacteria participated in nitrogen-cycling in legumes plant soil (Venturi and Keel, 2016). Enhanced specific activities of nitrogen-cycling enzymes accompanied by bacterial density-dependent behaviors in the rhizosphere soil suggest that AHL could be a control point in the complex process of rhizosphere nitrogen mineralization (DeAngelis et al., 2008; Lamers et al., 2012). Furthermore, AHL molecules can affect plant gene expression and physiological features such as growth rate, root development, and resistance to microbial pathogens (Scott et al., 2006; Ma et al., 2014). More recently, the presence of AHL signaling molecules in soil microbes can affect the production of extracellular hydrolytic enzymes, suggesting that AHLs may take part in population communications and co-exist behaviors, especially in plant and other complex environments (Crevelin et al., 2013; Jayaraman et al., 2014; Schikora et al., 2016; Venturi and Keel, 2016).

In wetland biosphere, bacterial communities associated with the rhizosphere of wetland plants seem to be unique because they are shaped by multiple plant and water factors and can form complex biofilms themselves. Furthermore, AHL often occurs in environments that are physically, biologically and chemically heterogeneous. Identification of AHL-producing microbes in rhizospheres of wetland plants could provide important information for understanding bacteria-bacteria and plant-bacteria interactions (Venturi and Keel, 2016). However, to this day, the diversity features of AHL signaling molecules in wetland sediments and their potential effects on plant health are still limited, and only a few studies have focused on mangrove plants (Zeng et al., 2014; Viswanath et al., 2015).

In order to fill this gap, we set out to investigate the diversity of cultivable AHL-producing rhizobacteria associated with the mangrove plant *Kandelia obovata* by (1) screening for AHL producers using reporter strains; (2) identifying positive bacterial strains by molecular sequencing; (3) profiling the chemical characteristics using thin-layer chromatography (TLC), ultra performance liquid chromatography, and mass spectrometry methods; and (4) drawing the taxonomical position of AHL-producing candidates by evolutionary tree construction. The aims of this work are to dissect the diversity profile of AHLs in the mangrove rhizosphere environment to better understand plant-microbial community associations on the micro-ecological level.

## Materials and Methods

### Chemicals and Reagents

Two AHL reporter strains were used in this study: *Agrobacterium tumefaciens* strains A136 (pCF372-PtraI-lacZ; detection of a broad range of C4-C12 AHLs) and KYC55 (pJZ372-traI-lacZ; detection of a broad range of C4-C18 AHLs). The two reporter strains were obtained as gifts from Dr. Zhigang Zhou (Chinese Academy of Agricultural Sciences, Beijing, China). *Pseudomonas aeruginosa* PAO1 (GDMCC No. 15692) and *Escherichia coli* DH5α (GDMCC No. 67878) from Guangdong Microbiology Culture Center of China (GMCC) were used as controls. X-Gal (5-bromo-4-chloro-3-indolyl-β-D-galactopyranoside) and AHL standards were obtained commercially from Sigma–Aldrich (St. Louis, MO, USA). Microbial media and salt solutions were purchased from Guoyao Co., Ltd. (Shanghai, China). Antibiotics, including tetracycline (4.5 μg/ml), spectinomycin (50 μg/ml), kanamycin (50 μg/ml), ampicillin (100 μg/ml), and gentamicin (40 μg/ml), were obtained from the National Vaccine and Serum Institute (NVSI, Beijing, China). They were supplemented into the growth medium whenever necessary. Methanol, acetonitrile, and other reagents with purity of >99.5% (analytical grade or chromatographical grade) were obtained from Shenggong Co., Ltd. (Shanghai, China).

### Sample Collection and Preparation

Naturally grown *K. obovata* mature fine branch roots with adhering rhizosphere soil were obtained from the Zhanjiang Mangrove National Nature Reserve (110008′ E, 20054′ N). It is the largest mangrove forest wetland reserve in China (about 20,279 ha), located along coastal areas of the Leizhou Peninsula at the southernmost tip of China between the South China Sea and the Tonkin Gulf, adjacent to Hainan Island. The Reserve is characterized by a subtropical oceanic monsoon climate, and the mean annual temperature is 22.9°C. The *K. obovata* root samples were collected at a depth of 10–15 cm (upper soil) using a spade and sterile scissors and placed in sterile polythene bags without air in coolers for transport to the laboratory. There are three trial sites to collect the samples, in each site three replicate rhizosphere soil were randomly collected to assemble a composite sample, which was used for bacterial screening. Three grams of root sample were added to 30 ml sterile saline (15 ppt) in a 50 ml sterile centrifuge tube, incubated with shaking for 30 min and then ultrasonicated for 30 s. Finally, the root was discarded and the soil suspension was separated into two 10 ml sterile tubes. One tube was processed for analysis of the microbial diversity and another tube was used for isolation of the bacterial strains. The environmental parameters temperature, pH, salinity, and conductivity were measured directly in the field, using a multiparameter probe (Multiparameter Display System Model 650, YSI, Yellow Springs, OH, USA).

### Diversity of Rhizobacteria

DNA was extracted using the PowerSoil kit (MoBio, Carlsbad, CA, USA) according to the manufacturer’s instructions. DNA samples were sequenced by Beijing Genomics Institute (BGI, Shenzhen, China) using the Illumina MiSeq platform with the primer set Gray28F–Gray519R as previously described (Lane, 1991). The obtained sequences were processed using Mothur (Schloss et al., 2009) according to the analysis pipeline described on the web site^1^. Sequences were analyzed for features including biodiversity, community, and taxonomy.

### Isolation of Bacterial Strains

The rhizosphere soil suspension was vortexed for 10 s, serially diluted and plated onto 2216E medium plates containing 1.2% agar. The formulae of 2216E medium (per liter) is peptone (5.0 g), yeast extract (1.0 g), ferric citrate (0.1 g), sodium chloride (19.45 g), magnesium chloride (8.8 g), sodium sulfate (3.24 g), calcium chloride (1.8 g), potassium chloride (0.55 g), sodium bicarbonate (0.16 g), potassium bromide (0.08 g), strontium chloride (34.0 mg), boric acid (22.0 mg), sodium silicate (4.0 mg), sodium fluoride (2.4 mg), ammonium nitrate (1.6 mg), disodium phosphate (8.0 mg), and agar (12.0 g). The plates were incubated at 28°C for 24–48 h. Unique colonies were selected based on color and morphology and transferred into 96-well plates with each well containing 100 μl LB (Luria–Bertani) liquid media. The plates were shaken slowly for 12 h. Colonies were mixed with glycerol (15%) and stored at -80°C for further analysis.

### Screening Test for AHL-Producing Strains

#### Preliminary Screening

*N*-acyl-homoserine lactones screen was detected using biosensors according to Chu et al. (2011) methods. Briefly, A136 and KYC55 were grown overnight at 28°C in 5 ml of LB medium. The reactivated rhizosphere isolates was cultured overnight at 28°C in 96-well plates. Seventy-five microliter of 5 × diluted A136 overnight culture were added to 75 μl of samples (activated rhizosphere isolates) in a 96-well plate and incubated at 28°C with constant shaking at 150 rpm before X-Gal (final concentration of 40 μg/ml) was added to each well. Plates were incubated overnight at 28°C and the strain with production of blue color after 24–48 h incubation was recorded as an AHL-producing strain. *P. aeruginosa* PAO1 was used as the positive control and *E. coli* DH5α as the negative control.

#### Second Screening

The potential AHL activity of the isolates was tested by well diffusion agar plate assays as described elsewhere (Zhu et al., 2001; Lv et al., 2016). Briefly, 5 ml overnight reporter strain culture was mixed with 50 μl X-Gal in 50 ml LB media containing 1% agar when the temperature of the LB was about 45°C. The mixture was plated and allowed to solidify before sterile filter paper circles (0.5 cm diameter) were attached to the LB surface at regular intervals. The putative AHL-positive bacterial strains identified in the preliminary screen were pipetted onto the filter paper. Positive AHL production was recorded as visible blue pigmentation after overnight incubation at 28°C. AHL production active against A136 was confirmed by streaking the test-isolate on LB agar plates supplemented with 40 μg/ml X-Gal. To detect if any of the isolates produced extracellular factors that could hydrolyze X-Gal and thus give a false-positive readout, isolates that produced a blue coloration in the patch test were retested on a plate containing only agar with X-Gal. Isolates producing blue coloration on these plates were considered false-positive results in the A136 or KYC55 assay and were scored as negative.

### Extraction of AHLs

The AHLs extract was adapted from the procedure of Krick et al. (2007). Each of the isolate cultures was grown 24 h in 200 ml volume with agitation at 200 rpm. The fermentation culture was centrifuged at 12,000 ×*g* for 10 min and extracted with an equal volume of acidified ethyl acetate (0.1% glacial acetic acid). The mixture was then shaken vigorously for 20 min. The ethyl acetate phase was removed, and the extraction was repeated. The combined extracts were evaporated in a rotary evaporator at 45°C. The residue was dried by nitrogen flow, reconstituted in 1 ml acidified ethyl acetate, transferred to HPLC glass vials and re-evaporated. The dry extracts were reconstituted in 100 μl acidified ethyl acetate and stored at -20°C.

### AHL Profiling Using TLC

The extracted samples together with the standards were profiled using C-18 reverse phase TLC plates (20 cm × 20 cm TLC aluminum sheets; RP-18 F254 S, Merck, Germany), and the chromatogram was developed with a methanol/water mixture (60:40) as described by Shaw et al. (1997). The developed active AHL spots were visualized by agar overlay bioassay using the bioreporter strain A136 on TLC plate. For the overlay, approximately 5 ml overnight mid-exponential A136 culture were added to 50 ml LB media containing 1% agar and 40 μg/ml X-Gal. The cultures were mixed and poured immediately over the developed TLC plates placed in sterile Petri dishes. The plates were incubated overnight and examined for the presence of respective color induction exhibited by the bioreporters. AHL chain lengths were rough calculated by comparing the *Rf* values and the shape of the spots with standard AHLs (*N*-butanoyl-L-homoserine lactone (C4-AHL), *N*-hexanoyl-L-homoserine lactone (C6-AHL), *N*-octanoyl-DL-homoserine lactone (C8-AHL), 3-hydroxy-*N*-homoserine lactone (3-OH-C8-AHL), *N*-decanoyl-DL-homoserine lactone (C10-AHL), N-dodecanoyl- DL-homoserine lactone (C12-AHL), and *N*-tetradecanoyl-DL-homoserine lactone (C14-AHL). Standard AHL molecules were obtained from Sigma–Aldrich (St. Louis, MO, USA) and used as controls.

### UPLC and MS Analyses of AHLs

*N*-acyl-homoserine lactones were identified by UPLC and liquid chromatography in tandem with mass spectrometry (LC-MS) detection methods. Samples were kept at 4°C until injection, and 10 μl of each sample were injected onto a reverse phase C18 core-shell column (Phenomenex Kinetex, Torrance, CA, USA) via a Thermo Electron Surveyor auto-sampler (Thermo Fisher Scientific, Waltham, MA, USA). Separation was obtained using a gradient of 0.1% acetic acid in water and 0.1% acetic acid in acetonitrile at a flow rate of 0.3 ml/min. The eluent was introduced into a TSQ Quantum Ultra Triple Stage Quadrupole mass spectrometer (Thermo Scientific) using electrospray ionization, and detection was achieved using multiple reaction monitoring (MRM) in positive ion mode. For identification of AHL molecules, the LC fractions were subjected to electro spray ionization tandem mass spectrometry (ESI-MS-MS; Ion Trap MS Esquire 3000 Plus) under positive ion conditions (Ortori et al., 2011). AHL molecules were detected by screening the samples for those precursor ions that gave rise to a fragment ion at m/z 102. All possible chain lengths ranging from 4 to 20 carbons were included in the method, and the potential to have a hydroxyl or ketone at the three position, with or without a single double bond in the chain, was also taken into account.

### Identification of AHL Bacteria by 16S rRNA Genotyping

Genomic DNA from AHL-positive strains was isolated using the UltraClean Microbial DNA Isolation Kit (MoBio Laboratories, Carlsbad, CA, USA). DNA was amplified using the universal primers 8F (5′-AGACTTTGATYMTGGCTCAG-3′) and 1512R (5′-GTGAAGCTTACGG(C/T)TAGCTTGTTACGACTT-3′) as previously described (Viswanath et al., 2015). Primers were obtained from the BGI (Shenzhen, China). The reaction mixture included 12.5 μl Reddy MixPCR master mix containing 1.5 mM MgCl_2_ and 0.2 mM of each deoxynucleoside triphosphate, 1 μl each of the forward and reverse primers, 1–2 μl of genomic DNA, and water to bring the total volume to 25 μl. An initial denaturing hot start of 4 min at 95°C was followed by 30 cycles of 94°C for 30 s, 55°C for 40 s, and 72°C for 70 s. The final extension step consisted of 20 min at 72°C, concluding the reaction. The PCR products were separated by electrophoresis on a 1.0% agarose gel and stained with ethidium bromide to confirm that an approximately 1,500 bp product was present. The purified PCR products were then sequenced by BGI (Shenzhen, China).

16S rRNA gene sequences were compared with those in the GenBank database using the basic local alignment search tool BLAST^2^. A ≥97% match of the unknown clone with the GenBank dataset was considered suitable identification at the species level. Similarity of 93–96% was accepted as genus-level identification (Stackebrandt and Goebel, 1994). The 16S rDNA sequences were aligned using the ClustalW multiple alignment package,^3^ and a consensus region covering all the sequences was selected for further analysis. The aligned sequences were subjected to phylogenetic tree construction using the neighbor-joining method provided by the MEGA4^4^ software package (Kumar et al., 2004). Maximum likelihood bootstrap analyses were carried out with 1,000 replicates.

### Statistical Analysis

The mean and standard deviations of environmental parameters were calculated using the Origin 8.1 software (OriginLab Corp., Northampton, MA, USA).

## Results

### Environmental Factors and Relative Diversity of Rhizobacteria

The sampling habitats were relatively similar in environmental parameters. The temperature, pH, salinity, and conductivity values were 23.4 ± 0.2°C, 6.36 ± 0.13, 6.90 ± 0.57‰, and 8.65 ± 1.44 ms^-1^, respectively.

The relative biodiversity of rhizobacteria was shown in **Supplementary Figure S1**. A total of 15 phyla were identified from the tested samples, and the top-five phyla (Proteobacteria, Chloroflexi, Bacteroidetes, Planctomycetes, and Actinobacteria) contributed up to more than 90% of the total quality reads (**Supplementary Figure S1A**). Among the Proteobacteria, Gamma-proteobacteria, Delta-proteobacteria, and Alpha-proteobacteria were the most abundant classes, which occupied 64.4, 25.8, and 7.7% percentage (**Supplementary Figure S1B**). The Candidate division WS3, Gemmatimonadetes, Actinobacteria, Deferribacteres, and Spirochaetae as the minorities and comprised 2.0, 1.4, 0.9, 0.7, and 0.5% of the total population, respectively. The unidentified species is about 2.5% (**Supplementary Figure S1A**). When sequences were assigned to the taxonomic rank of “order,” 10 taxonomic groups were found to make up 88.9% of the total quality reads. Vibrionales (43.8%) and Desulfobacterales (15.7%) were the two most abundant, followed by Anaerolineales (5.5%), Alteromonadales (5.5%), Xanthomonadales (4.5%), Myxococcales (3.6%), Rhodospirillales (3.5%), Chromatiales (3.1%), Cytophagales (1.8%), and Oceanospirillales (1.5%). The less abundant taxa (near 1%) were Rhizobiales (1.26%), Desulfuromonadales (1.07%), Deferribacterales (1.01%), Rhodobacterales (0.98%), and Desulfarculales (0.79%; **Supplementary Figure S1C**).

### Screening for AHL Producers

A total of 1200 cultivable strains were isolated from rhizosphere soil and screened for AHL activity. Of these isolates, 300 were identified as AHL-producing candidates active with at least one of the two reporter strains (A136 or KYC55) in the preliminary screening. After verification by second screening and removal of false-positives (the rate was about 20%), 184 bacterial strains gave rise to a positive signal as blue color zones with both reporter strains (A136 and KYC55) were observed. The activity of positive isolates was recorded as either strong (++) or weak (+) based on the color intensity produced by the reporter strains. The data obtained are summarized in **Table 1** (part A). In total, AHL-producing bacteria represented 15.33% (184/1200) of all cultured bacteria isolated from *K. obovata* rhizosphere soils.

**Table 1:** *N*-acyl-homoserine lactone (AHL) profiling of 24 representative rhizobacteria isolated from the mangrove rhizosphere environment.

Part A			Part B			
Strain No.	A136	KYC55	OTU No.	Closest cultivated bacteria	ID at16S-rRNA gene cocus	Potential AHL compounds^∗^
Strain 1	++	++	OTU1	*Enterobacter* sp.	99%	C12-, C8-OH, C6-


Strain 2	++	++	OTU2	*Pasteurella pneumotropica*	100%	C12-, C10-, C8-OH, C6-


Strain 3	++	++	OTU3	*Photobacterium rosenbergii*	99%	C12-, C8-, C8-OH


Strain 4	++	++	OTU5	*Vibrio fluvialis*	99%	C8-OH


Strain 5	++	++	OTU6	*Gallaecimonas* sp.	99%	C10-


Strain 6	+	++	OTU8	*Staphylococcus xylosus*	99%	C10-


Strain 7	+	+	OTU9	*Vibrio sinaloensis*	99%	C8-OH


Strain 8	++	++	OTU10	*Brachybacterium paraconglomeratum*	99%	C14-, C12-, C8-OH


Strain 9	+	+	OTU11	*Bacillus aerophilus*	99%	C12-, C10-, C8-OH


Strain 10	++	++	OTU12	*Vibrio communis*	100%	C12-, C10-, C8-OH


Strain 11	+	++	OTU13	*Staphylococcus saprophyticus*	100%	C14, C12-, C8-OH
Strain 12	+	–	OTU14	*Photobacterium* sp.	99%	C12-
Strain 13	+	+	OTU15	*Bacillus* sp1.	99%	C14-, C12-
Strain 14	++	+	OTU17	*Bacillus aquimaris*	99%	C8-
Strain 15	++	++	OTU18	*Vibrio* sp.	99%	C14-, C12-, C8-OH
Strain 16	+	+	OTU19	*Bacillus cereus*	99%	C8-
Strain 17	+	+	OTU20	*Paenibacillus* sp.	100%	C12-, C8-
Strain 18	++	++	OTU21	*Paracoccus* sp.	100%	C12-, C10-, C8-, C6-
Strain 19	++	++	OTU22	*Psychrobacter* sp.	99%	C14-, C12-, C8-
Strain 20	+	+	OTU23	*Bacillus* sp2.	99%	C8-
Strain 21	++	–	OTU24	*Alteromonas* sp.	99%	C10-
Strain 22	++	++	OTU26	*Acinetobacter* sp.	99%	C12-
Strain 23	++	++	OTU27	*Pseudomonas chlororaphis*	99%	C14-, C12-, C10-
Strain 24	++	++	OTU28	*Pseudomonas aeruginosa*	98%	C14-, C10-, C8-OH


++, Strong colouration; +, Weak colouration; -, No colouration. ^∗^Detection of AHL compounds using bioreporter strain A136 as an agar overlay on thin-layer chromatography (TLC) plates. AHL identified by the spot shape and corresponding *Rf* value. Part A: AHL activity detected from plate bioassays using bioreporters (A136 and KYC55). Part B: Identification and compounds information of AHL-producers.

### Identification of AHL-Producing Bacterial Strains by 16S rRNA Sequencing

The 16S rRNA gene sequences of these 184 bacterial isolates were aligned to the NCBI database using BLAST. BLAST results showed that 82 of the total belonged to the class Gamma-proteobacteria, 45 to the class Alpha-proteobacteria, 36 to the phylum Firmicutes, and 17 to the phylum Actinobacteria. After dereplication analyses, 24 AHL-producing rhizobacterial representatives were chosen from the candidates for taxonomical identification and AHL profiling studies. Most of the representative isolates shared 99% sequence similarity and five of them had a 100% sequence similarity with their respective reference strains (**Table 1**, part B). These 24 bacterial strains represented four different bacterial groups, 13 belonging to Gamma-proteobacteria, 9 to Firmicutes, 1 to Alpha-proteobacteria, and 1 to Actinobacteria. The bacterial sequences have been submitted to GenBank Database and the accession number is KX941451–KX941474.

**FIGURE 1:**
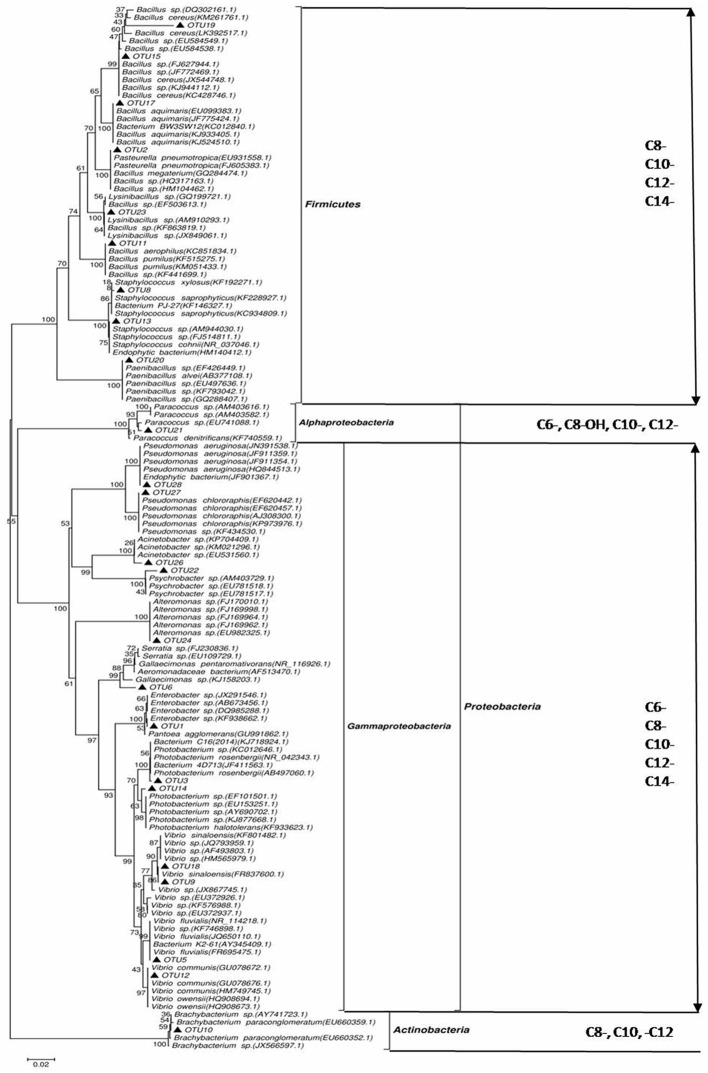
**Phylogenetic tree of *N*-acyl-homoserine lactone (AHL)-producing rhizobacteria based on neighbor joining clustering and species identification by 16S rRNA gene sequence alignment to the NCBI GenBank database.** Phylum taxa appear on the right, with the external distribution made by class and the internal distribution made by genus. ▲, isolates that induced AHL activity detected by the A136 indicator strain. Bootstrap probabilities are indicated at branch nodes (values under 50% were excluded). The bar represents three substitutions per 100 nucleotide positions. GenBank accession numbers deposited in the NCBI database are given for partial 16S rRNA sequences of all isolates.

A phylogenetic tree based on the 16S rRNA gene sequences was constructed by neighbor joining clustering for the 24 AHL-positive bacterial strains. The AHL producers were classified into four different bacterial clusters, namely Gamma-proteobacteria, Actinobacteria, Alpha-Proteobacteria, and Firmicutes (**Figure 1**). In the Gamma-proteobacteria family, most of the positive strains were related to the genus *Vibrio* sp., *Enterobacter* sp., *Alteromonas* sp., *Photobacterium* sp., and *Pseudomonas* sp. Six AHL-producing members of the Firmicutes group were closely related to the genus *Bacillus* sp., two to *Staphylococcus* sp., and only one to the genus *Paenibacillus* sp. One strain (OTU21), for which AHL activity was only detected by the bioluminescent reporter strain A136, was assigned to the *Paracoccus* genus of Alpha-proteobacteria. 16S rRNA gene sequence analysis indicated that OTU10 shared the greatest similarity to the *Brachybacterium* sp. (99% similarity), forming a cluster in Actinobacteria, which was closely related to *B. paraconglomeratum*. Remarkably, this strain belongs to a genus that has never been reported to synthesize AHLs.

### Profiling of AHL Molecules

**FIGURE 2:**
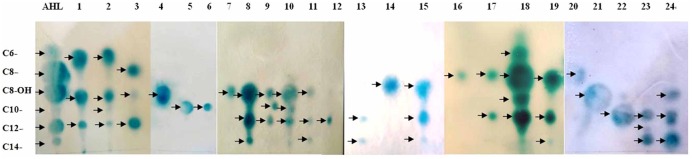
**Analysis of AHLs from supernatant extracts with the sensor strain.** AHLs extracted from cell-free culture supernatants were separated by thin-layer chromatography (TLC) and detected using an overlay of agar seeded with *A. tumefaciens* 136. Line AHL are the QS standards (arrows point to C6-, C8-, OH-C8-, C10-, C12-, and C14-AHL, respectively). Lines 1–24 represent the sample extracts. The strain names were: *Enterobacter* sp., *Pasteurella pneumotropica*, *Photobacterium rosenbergii*, *Vibrio fluvialis*, *Gallaecimonas* sp., *Staphylococcus xylosus*, *Vibrio sinaloensis*, *Brachybacterium paraconglomeratum*, *Bacillus aerophilus*, *Vibrio communis*, *Staphylococcus saprophyticus*, *Photobacterium* sp., *Bacillus* sp1., *Bacillus aquimaris*, *Vibrio* sp., *Bacillus cereus*, *Paenibacillus* sp., *Paracoccus* sp., *Psychrobacter* sp., *Bacillus* sp2., *Alteromonas* sp., *Acinetobacter* sp., *Pseudomonas chlororaphis*, and *Pseudomonas aeruginosa*.

Reverse phase TLC was utilized to determine the signal molecule profiles of the 24 positive strains. A diverse range of AHL profiles was observed among the mangrove rhizobacteria. Most of the isolates produced more than one active compound, which included both 3-unsubstituted acyl-AHLs and 3-hydroxy-substituted acyl-AHLs (**Figure 2**). Among the AHL substances, the dominant signals belonged to mid length compounds ranging from C8- to C12-. The most common AHL variant produced by the 24 isolates was C12-, produced by 17 (70.8%) of the strains in liquid culture, followed by C8- or OH-C8-, detected in 12 (50.0%) of the strains. A broad range of AHL molecules, including short- and long-chains with different substituted side chains (C6–C12 or C8–C14), was detected in some strains. For example, the *Bacillus aerophilus* (OTU11) and *Vibrio communis* (OTU12) produced triple AHL profiles that had three circular spots with *Rf* values of 0.52, 0.33, and 0.18, representing C8-OH, C10-, and C12-AHL, respectively. In addition, *Bacillus* sp3. (OTU15) and *Pasteurella pneumotropica* (OTU2) produced a double and a quadruple spot in the TLC plate, respectively (**Table 1**, Part B).

### UPLC and LC-MS Analysis of AHLs

**FIGURE 3:**
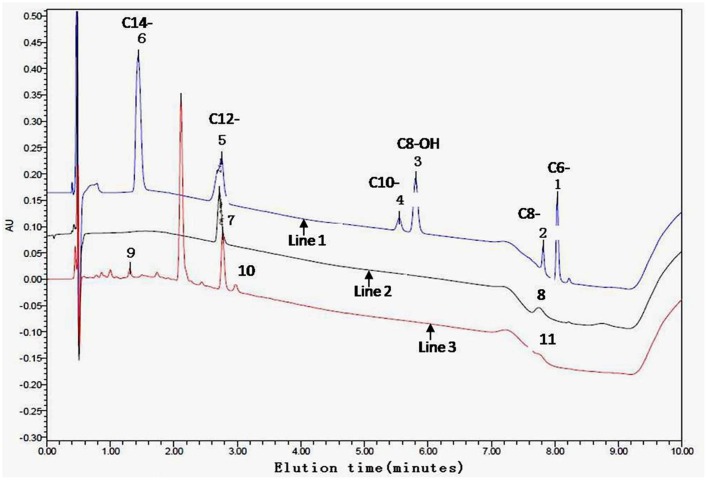
**Ethyl-acetate extracted culture supernatant analyzed by ultra performance liquid chromatography (UPLC).** A gradient from 30 to 100% acetonitrile was applied at a flow rate of 0.3 ml/min in 0.5 min intervals following an isocratic flow of 0.2 min with the initial composition. The controls (line 1) used were mixtures of six AHL standards (C6-, C8-, C8-OH, C10-, C12-, and C14-) with the following retention times: peak 1 is C6-, peak 2 is C8-, peak 3 is C8-OH, peak 4 is C10-, peak 5 is C12-, and peak 6 is C14-AHL. UPLC showing the presence of at least one AHL compound in the tested samples 17 (line 2) and 15 (line 3) as examples. Peaks 7 and 10 probably are C12-, peaks 8 and 11 are C8-, and peak 9 is C14-AHL, respectively.

*N*-acyl-homoserine lactone-positive strain extracts on the TLC plate were spiked and further analyzed by UPLC. Peaks of samples and AHLs standards were combined at a retention time of 10.0 min. A calibration curve was prepared with standard AHLs and used to estimate the AHL amount in the extracts at various time points. The controls were mixtures of six AHL standards (C6, C8, C8-OH, C10, C12, and C14) with their retention times. For example, for samples 15 (OTU18) and 17 (OTU20), peaks 7 and 10 are likely C12-AHL, peaks 8 and 11 are C8-AHL, and peak 9 is C14-AHL (**Figure 3**; **Table 1**, part B).

**FIGURE 4:**
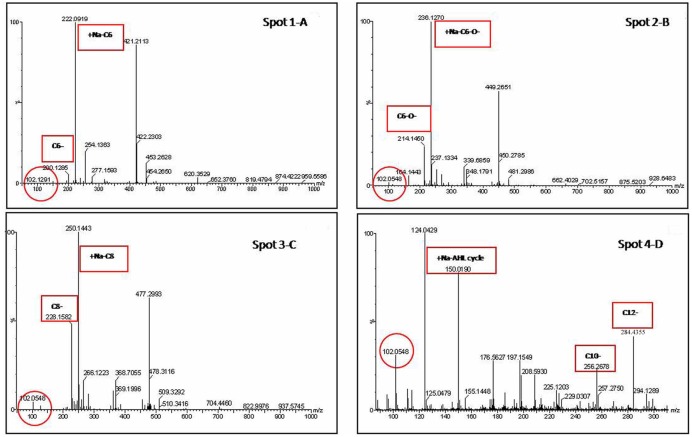
**Extracted ion chromatogram of the m/z 102 fragment (a characteristic of the homoserine lactone ring) of AHLs produced by representative strain (OTU2) recovered from preparative TLC.** Identification of the AHL in the extract was determined from the mass spectrometry (MS) spectra by comparison with the corresponding AHL standards. For example, the MS data for the spot 1 **(A)** extract indicates the presence of a characteristic lactone fragment at m/z of 102 and the molecular ion peak at m/z of 200 or 220 [Na^+^], suggesting the AHL to be C6-AHL. Accordingly, The spot 2 **(B)**, spot 3 **(C)**, and spot 4 **(D)** are C6-O-, C8-, and (C10- or C12-) AHL, respectively.

Liquid chromatography in tandem with mass spectrometry (LC-MS) of AHLs contains a molecular ion [M+H]^+^ or [M+Na]^+^ peak and a characteristic lactone fragment peak at m/z of 102. Based on these two analyses (TLC and LC-MS), the corresponding AHL substances of these AHL molecules were identified. For example, LC-MS data for the OTU2 extract show the presence of a characteristic lactone fragment at m/z of 102 and the molecular ion peak at m/z of 200 or 222 [Na^+^], suggesting the AHL to be C6-AHL (spot 1; **Figure 4A**). Accordingly, spot 2 (B), spot 3 (C), and spot 4 (D) are C6-O-AHL, C8-AHL, and C10- or C12-AHL, respectively. **Figure 4** shows the LC-MS spectra acquired from chromatographic runs of extracts with chromatographic peaks that further confirm the patterns observed on the TLC plates. The LC-MS analysis further revealed that the isolates produced AHLs of varying acyl chain lengths ranging from 6 to 14 carbons. Some signal-producing isolate synthesized more than one type of AHL, and multiple genera/species could produce the same AHL. The dominant signal detected in the root community was from medium AHLs.

## Discussion

### Overview of Bacterial Diversity

The dominant native mangrove species in Leizhou Reserve are *K. obovata*, *Aegiceras corniculatum*, and *Avicennia marina*. In the present study, we focused on *K. obovata* and found that prokaryotic communities in mangrove roots were dominated by Proteobacteria, Chloroflexi, Bacteroidetes (previously Cytophaga–Flexibacter–Bacteroides), Planctomycetes, and Actinobacteria (**Supplementary Figure S1A**). The observation was consistent with previous reports (Andreote et al., 2012; Hong et al., 2015; Zhang et al., 2015). Among the Proteobacteria, Gamma-proteobacteria, Delta-proteobacteria, and Alpha-proteobacteria were the three most abundant classes in our sampling sites (**Supplementary Figure S1B**), in agreement with previous findings in *Aegiceras corniculatum*, *Avicennia marina*, and *Sonneratia caseolaris* (Yang et al., 2014; Alzubaidy et al., 2016; Chen et al., 2016). These results suggest that Alpha-, Gamma-, and Epsilon-proteobacteria are prevalent in mangrove plants. Beta-, Zeta- and Epsilon-proteobacteria comprise only 2.02% of the total quality reads, which is lower than Liang et al. (2007) reported result (16%). Biogeography (distinct soil depths and locations) may be a major determinant factor that influences bacterial structure. In addition, anthropogenic interference may contribute to this difference, because various degrees of human activities (urbanization, pollution, and aquaculture) have different effects on environmental competitiveness and co-shaping the microbial communities (Marcial et al., 2008; Basak et al., 2015; Chakraborty et al., 2015).

At order level, Vibrionales (belongs to Gamma-proteobacteria) was the most predominant group and approximately 43.8% of the OTU sequences were classified in it (**Supplementary Figure S1C**). The abundance of Vibrionales was much higher in *K. obovata* rhizosphere samples than bulk sediment samples (Yin et al., 2016). The possibility of this distribution pattern of Vibrionales may be attributed to the contribution of root influence. Around the mangrove habitat, root secrete can attract some heterotrophic bacteria and most of them were Vibrionales, Actinomycetales, and Bacillales (Dias et al., 2009). Desulfobacterales (belongs to Delta-proteobacteria) sequences represent the second largest fraction (15.7%) of total quality reads at class level, and the abundance of Desulfobacterales is higher compared with that in published literature (Zhang et al., 2016). The Desulfovibrionales has been reported to be able to adapt to or tolerate to environmental stresses, such as low salinity or low sulfate supply (Kuever et al., 2005; Leloup et al., 2006), heavy metals contamination (Cabrera et al., 2006), and anthropogenic activities (Miralles et al., 2007). The presence of such organisms and the shifts observed in these groups are indications that the effects of environmental conditions on mangrove functioning might exert selection pressure on the sulfate- and sulfite-reducing process. In addition, Desulfovibrionales has a more efficient nutrient uptake and reduced energy utilization in oligotrophic environment (Varon-Lopez et al., 2014), partly explaining their distinct distribution patterns. It is worthwhile to note that some fluctuates appeared in non-abundant species (Anaerolineales, Alteromonadales, Xanthomonadales, Myxococcales, Rhodospirillales, and Chromatiales), hinting the possibility of terrigenous influence in the community variation.

### Relative Occurrence of AHL-Producers in the Rhizosphere Environment

This work found the presence of AHL-producers among the *K. obovata* rhizobacteria, comprising 15.33% of a total of 1200 cultivable bacterial strains. In this study, the pH value in rhizosphere environment ranged from 6.23 to 6.49, making the mangrove soils suitable environment for AHL substances or their producers. Furthermore, the high percentage of AHL-producing strains isolated in the present study could indicate that AHLs are strategically used in the rhizosphere niche to achieve competitive advantages, at least in the fluctuating mangrove habitats exposed to tidal cycles (Krumme et al., 2012). More detailed studies on the presence of AHL activity in cultivable bacteria in mangrove plants should be carried out and would likely add valuable information to the elucidation of the ecological importance of AHL-mediated micro-environmental processes in the wetland ecosystem (Romero et al., 2011).

Among the 24 AHL-producing rhizobacterial representatives, 16S rRNA gene sequences exhibited greater than 98% similarity to known species belonging to the genera *Bacillus*, *Photobacterium*, *Gallaecimonas*, *Vibrio*, *Staphylococcus*, *Alteromonas*, *Paracoccus*, *Brachybacterium*, *Pseudomonas*, *Acinetobacter*, *Enterobacter*, and *Psychrobacter* (**Table 1**). The Alpha- and Gamma-proteobacteria represented approximately 60% of these AHL-producing bacteria. The fact that most of these AHL-producing bacteria are Proteobacteria is in agreement with the up-to-date reports that AHL synthesis has only been found in the members of these three phylogenetic groups (alpha-, beta-, and gamma-) so far (Gray and Garey, 2001; Manefield and Turner, 2002). The remaining strains belong to Firmicutes (*Paenibacillus* sp.) and Actinomyces (*Brachybacterium* sp.). Some AHL producers isolated in this study belong to known AHL-producing genera but other strains were detected for the first time, including *Brachybacterium paraconglomeratum* (belongs to the Dermabacteraceae family) and *Acinetobacter* sp., which implied mangrove root habitat as a reservoir for AHL screen to be explored. Interestingly, we identified three Gram-positive bacteria (*Staphylococcus* sp., *Bacillus* sp., and *Brachybacterium* sp.) that could produce AHLs. Since presently known AHL producers are Gram-negative bacteria, we speculated that genetic events (gene flow or horizontal gene transfer) from certain Gram-negative bacterium/bacteria might occur to confer the AHL productivity to Gram-positive individual. Deeper sequence analysis of this strain will allow these hypotheses to be assessed and an ecological interpretation of this functional versatility to be proposed (Biswa and Doble, 2013).

It is worth noting that compared to the composition of total microbial structures in rhizobacteria, five phyla (Proteobacteria, Chloroflexi, Bacteroidetes, Planctomycetes, and Actinobacteria) dominated the strains, comprising more than 90% percent of the total OTUs (**Supplementary Figure S1A**). In agreement with this, the cultivated root-associated strains producing AHLs seem to be more frequently clustered with these bacteria which are commonly found in the rhizosphere holobiont. This observation suggests that AHL producers mainly come from the predominate taxa.

### The Diversity and Characterization of AHLs

Thin-layer chromatography coupled with AHL bioreporters gave a rapid and directly visible system for the detection of AHL molecules. UPLC and LC-MS further confirmed the inducing signal to come from AHL substances (**Figures 3** and **4**). The TLC plate showed more than one AHL molecule in each positive isolate and most frequently identified medium- or long-chain AHLs (such as C8-, C10-, and C12-; **Figure 2**). The broad AHL spectrum suggests that the root microbial community has the capacity to synthesize multiple signals. This observation also corresponds with the profile of AHLs identified in the floccular sludge community (Tan et al., 2014). In addition, our results are consistent with a previous report that AHL-producing bacteria often produce long-chain rather than short-chain AHLs (Wagner-Dobler et al., 2005). This could be because short-chain AHLs are known to degrade more rapidly in saltwater environments (Hmelo and Van Mooy, 2009). Furthermore, based on the pH sensitivity of AHL molecules, low or high soil pH may be negative selection factors for short-chain AHL-producers. This suggests a dominance of longer-chain AHLs associated with the mangrove root species, where moderate acid pH predominates. In addition to the above-mentioned points, secreting medium- and long-chain AHL molecules could be an intelligent behavior of bacteria balancing the energy expended on self-preservation and ecological function (Winans, 2006; Jia et al., 2016). This hypothesis still needs to be further confirmed in future studies.

From the TLC results, we saw that isolates varied in the relatively quantity of AHL production (**Table 1**, part B). It has been suggested that there could be more than one AHL-regulatory system, and that more than one signal molecule could be produced by AHL synthases (Viswanath et al., 2015). The high heterogeneity in AHL production is in agreement with the high genetic diversity bacterial species. Though some bacterial isolates representing different genera produce a similar group of AHL molecules; for example, the major products among the representative Proteobacteria as well as Firmicutes are medium-chain AHLs (C8–C12), the roles of these similar AHLs in the phenotypic regulation may differ between different strains. This might be due to the procurement of homologous AHL genes by means of horizontal gene flow. Thus, the production of similar types of AHL molecules in different genera might help interspecies communication in the natural environment where mixed communities are often present (Viswanath et al., 2015).

In previous studies, researchers found that AHL profiles were not strictly conserved at the genus or species levels (Zimmer et al., 2014; Rajput et al., 2016). Hence, some phylogenetically distant species exhibited similar AHL profiles. Several studies can provide plausible explanations for these complex patterns of AHL production. At the molecular level, the amino acid sequences of AHL synthases sometimes are more dissimilar within one species than between distinct species (Gray and Garey, 2001). At the ecological and evolutionary level, multiple AHL synthase homologs or multiple *luxI/luxR* determinants in a bacterium could be acquired independently (D’Angelo-Picard et al., 2005). In addition, such heterogeneity within AHL profiles may result from a selective pressure that favors distinct molecular languages at the subspecies level, especially when related organisms share common ecological niches such as the rhizosphere (Steidle et al., 2001).

It is worth noting that we identified some AHL-producing isolates (for example, *Vibrio* sp. and *Aeromonas* sp.) that also have AHL-degrading genes or regulators using the SigMol tool (Rajput et al., 2016). The coupling of AHL synthesis and degradation in the same bacterium has previously been noted for *Agrobacterium tumefaciens*, which produces and degrades 3-oxo-C8-AHL via a lactonase encoded by *AttM* that is activated by starvation signals and stress alarmone (p)ppGpp (Zhang et al., 2004). Haudecoeur and Faure (2010) further pointed out that *A. tumefaciens C58* exhibits a fine control of biosynthesis and biodegradation of O-C8-AHL by lactonases *AttM* and *AiiB.* Similarly, a marine *Shewanella* strain that produces AHLs in the late exponential phase degrades its long-chain AHLs via both lactonase and acylase/amidase activities (Tait et al., 2009). Indeed, organisms known to be AHL producers or quenchers, such as members of Proteobacteria and Bacteroidetes phyla, have recently been expanded to also include Cyanobacteria, Acidobacteria, and Archaea from a diverse range of environments (Chan et al., 2011; Tan et al., 2015). So why do some strains of the same species act as AHL producers or degraders in different specified circumstances? In the rhizosphere habitat, extremely diverse microbial species co-exist, and the co-occurrence of AHL and anti-AHL activities is likely common (D’Angelo-Picard et al., 2005). A preliminary molecular analysis in our group suggests that multiple AHL-producing and AHL-quenching genes are present in the same marine bacterium from draft genome sequences (data not shown), supporting the co-occurrence of AHL and anti-AHL activities in the bioreactor. As for the role of these bifunctional bacteria, we speculate that they modulate signaling at the community level and could potentially be used as bio-controllers targeting AHL-regulated functions, especially in complex environments (D’Angelo-Picard et al., 2005). For instance, as suggested earlier, the lack of short-chain AHLs in the root may not be due to the absence of producer strains but more likely a consequence of specific AHL-degrading activity by the community.

### The Connection between the Root and AHLs or AHLs-Producers

Quorum sensing signals play an important role in the ecological interactions between bacteria and their eukaryotic hosts. In terrestrial plants, QS can control the virulence of pathogens to tobacco (Chevrot et al., 2006; Lang et al., 2016), and quorum-quenching bacteria capable of promote potato (*Solanum tuberosum*) growth (Cirou et al., 2007). In aquatic environment, many work have also demonstrated that AHLs can regulate the plant hosts’ ecological behaviors, including red algae biofouling (Manefield et al., 1999), green seaweed attachment (Joint et al., 2007), sponge release (Taylor et al., 2004), phycosphere carbon cycle (Huang et al., 2016), and marine snow alkaline phosphatase biosynthesis (Jatt et al., 2015). Similar phenomena were observed in the mangrove habitat; a conceptual model suggested that bacterial density-dependent behaviors might play a role in regulating mangrove soil nitrogen-cycling (DeAngelis et al., 2008). Density-dependent QS provides an interesting potential checkpoint for enzymes mediating organic nitrogen depolymerization and rhizosphere nitrogen mineralization (DeAngelis et al., 2008). These findings indicate that QS is an essential ecological signal modulator between plant and microbes.

This study has initiated steps toward understanding the role of AHL-producing bacteria associated with mangroves. Mangrove soil functions are closely related to bacterial activities, some plant-growth-promoting or biocontrol bacteria are key regulators for host healthy. Previously, Joseph and Phillips (2003) deemed it is conceivable that bacterial AHLs may represent a plant-microbe interaction in which both plant and root-associated bacteria benefit from the production of QS signals in the rhizosphere. It is possible to affect soil function by controlling bacterial activity in the soil via QS (Huang et al., 2013; Zeng et al., 2014). The study of the distribution and diversity of AHL-producers greatly increases our knowledge about potential cell-cell communications in natural niches and provides valuable information for the *in situ* control of bacterial activities. In this current work, 24 AHL-producing genera, such as Alteromonas, Vibrio, and Pseudomonas were identified in wetland samples using culture-dependent methods. Functionally, these putative AHL-producers could be classified into four groups according to their closest matches to indicate their possible relationships with the host plant: plant growth promotion, pathogenesis, nitrogen-fixing, and bioremediation (Parte et al., 2010). *Alteromonas* sp. and *Photobacterium* sp. were the predominant cultivable AHL-producers in the rhizosphere of wetland plants. Species from these two genera, such as *A. veronii* and *P. rosenbergii*, may act as beneficial rhizobacteria (plant growth promotion rhizobacteria, PGPR) to promote growth of plant (Ortiz-Castro et al., 2009). PGPR are able to stimulate plant growth by direct or indirect mechanisms, like production of phytohormones, mineralization of organic matter, and competing with pathogens (Ortiz-Castro et al., 2009). Some *Pseudomonas* and *Vibrio* species were considered to be potential pathogen to wetland plants (Pollumaa et al., 2012). Previously, QS has been shown to control Ti plasmid transfer in tumor-forming *Agrobacterium* spp. and virulence factor production in soft-rotting *P. carotovorum* (Pollumaa et al., 2012; Lang et al., 2016). Communication via diffusible signaling molecules between plants and bacteria has been proposed to be one of the important regulators in symbiotic behaviors (Scott et al., 2006).

In order to address the complete picture of AHL-producers and their ecological functions in the mangrove ecosystem, reporter strains in combination with non-cultivable approaches targeting AHL genes warrant further investigation (Viswanath et al., 2015). Another question worth pursuing in the future is the exploration of the parallel existence of AHL-producers and AHL-degraders in the rhizosphere environment and their roles in matter cycling (C, N, and P) in mangrove plants.

## Conclusion

This study confirms the existence of multiple AHL producers in the *K. obovata* rhizosphere environment, which provided novel information concerning the profiles of AHL signals in mangrove-associated bacteria. It is helpful for us to better understand the complex relationship between mangrove plants and microbes from chemical ecological perspective. Our work also speculated that mangrove rhizosphere bacteria might act as a candidate reservoir for identification new AHL-producers. In future, an urgent requisite aspect is to arbitrate the crucial role of AHLs associated traits of the rhizosphere bacteria, particularly their impact via signal mechanism on mangrove ecosystem, which are currently under way in our laboratory.

## Author Contributions

ZM and YL performed the experiment and write the manuscript. GL and HJ deal with the samples and analyzed the data. JZ and ZC provided funding and experimental conditions, and edited the manuscript.

## Conflict of Interest Statement

The authors declare that the research was conducted in the absence of any commercial or financial relationships that could be construed as a potential conflict of interest.

## Footnotes

1 http://www.mothur.org/wiki/MiSeq_SOP

2 http://www.ncbi.nlm.nih.gov/BLAST

3 http://www.mbio.ncsu.edu/bioedit/bioedit.html

4 http://www.megasoftware.net/mega4/mega.html

## Supplementary material

FIGURE S1 **Taxonomic composition of the root-associated microbiome of *K. obovata* from rhizosphere soil samples based on 16S rRNA sequencing. (A)** Bacteria diversity at phylum level; **(B)** classes of Proteobacteria; and **(C)** the bacteria biodiversity at order level. The sequencing experiments were repeated three times.
