# Supplementary figures and images for: Diverse Profiles of AI-1 Type Quorum Sensing Molecules in Cultivable Bacteria from the Mangrove (Kandelia obovata) Rhizosphere Environment
Source: Front Microbiol. 2016 Dec 5;7:1957. doi: 10.3389/fmicb.2016.01957 (PMC5136546; doi:10.3389/fmicb.2016.01957)

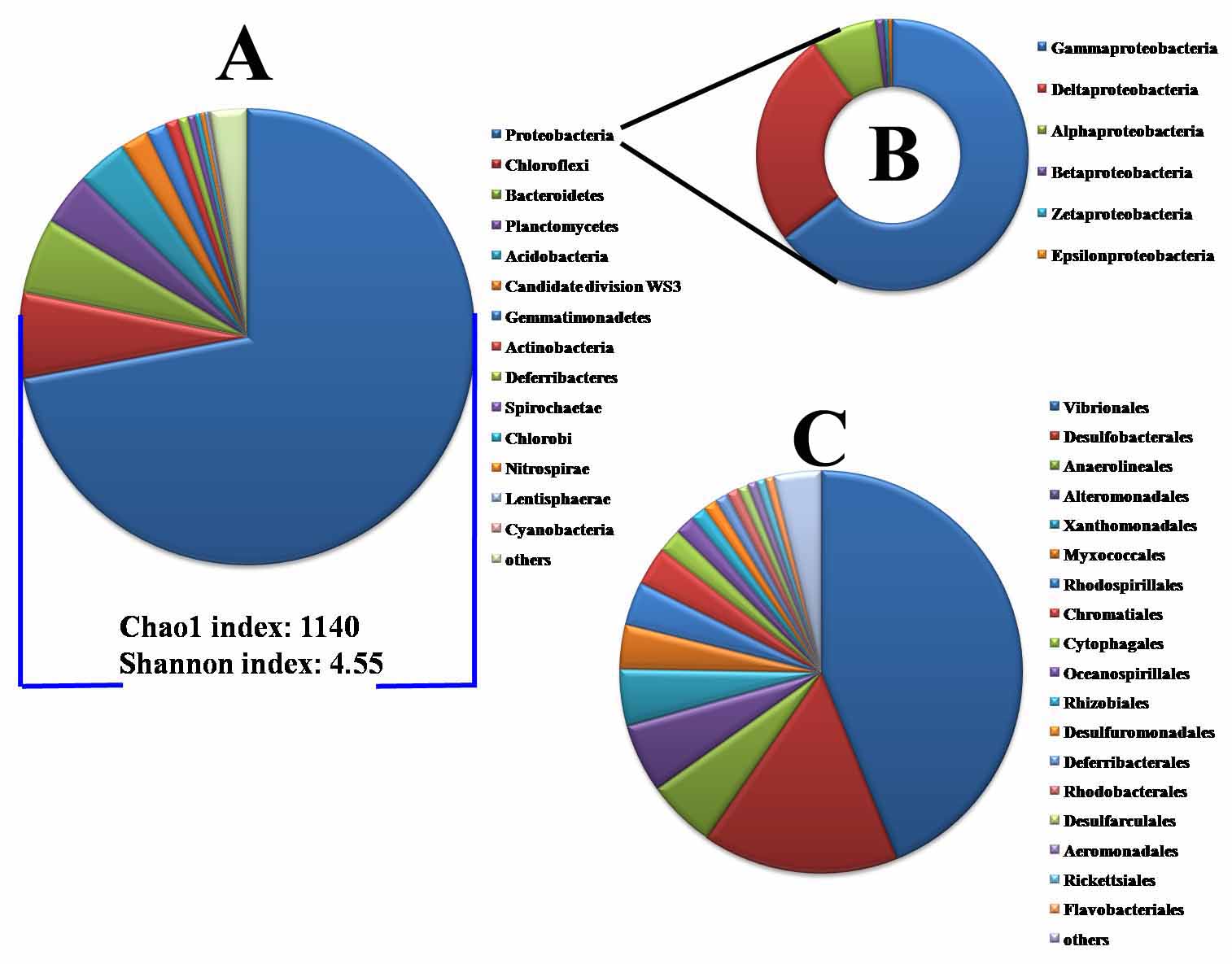

Supplement: FIGURE S1 — Taxonomic composition of the root-associated microbiome of K. obovata from rhizosphere soil samples based on 16S rRNA sequencing. (A) Bacteria diversity at phylum level; (B) classes of Proteobacteria; and (C) the bacteria biodiversity at order level. The sequencing experiments were repeated three times. [file Image_1.JPEG]
